# Nanostructural Characterization of Gold Nanorod Assemblies in Hexagonal Liquid Crystalline Template

**DOI:** 10.1021/acs.langmuir.6c03954

**Published:** 2026-09-10

**Authors:** Olga Kaczmarczyk, Dominika Benkowska-Biernacka, Julita Wesołowska, Mateusz Grzybowski, Amelia Maj, Katarzyna Matczyszyn, Andrzej Żak

**Affiliations:** † Institute of Advanced Materials, Wroclaw University of Science and Technology, 27 Wybrzeze Stanislawa Wyspianskiego St., 50-370 Wroclaw, Poland; ‡ Laboratory of Microscopic Imaging, Maj Institute of Pharmacology Polish Academy of Sciences, CEPHARES, 12 Smetna St., 31-343 Krakow, Poland

## Abstract

Exploiting the unique anisotropy and fluidity of liquid crystals (LCs) enables the design of advanced hybrid nanomaterials for photonic applications. The intrinsic long-range order of the LCs matrix can act as a structural template, directing nanoscale dopants into well-defined geometrical configurations and enabling precise control over nanoparticle distribution. Here, we use an inverted hexagonal phase (H_2_) of sodium bis­(2-ethylhexyl) sulfosuccinate (AOT), *p*-xylene, and a water dispersion of gold nanorods (AuNRs) to fabricate well-ordered nanomaterial assemblies. Scanning transmission electron microscopy of thin films reveals AuNR chains extending up to 30 μm in length with a mean interparticle spacing of about 5 nm and a 2D order parameter *S*_2D_ = 0.96 ± 0.10. Correlative multiphoton and polarizing microscopy demonstrate a uniform arrangement of AuNRs within the central region of the sample thickness, particularly at the domain boundaries. The performed nanostructural characterization suggests the ordering mechanism and highlights the impact of anchoring forces on nanorod ordering and microstructure formation in the bulk sample.

## Graphical abstract

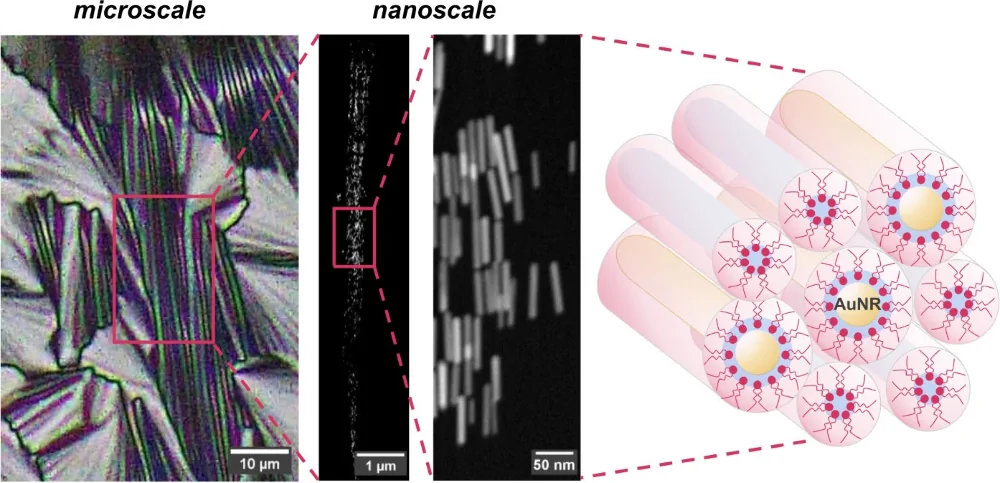

## Introduction

The exceptional properties of liquid crystals (LCs),^1,2^ which combine the isotropic characteristics of liquids with the anisotropy of crystals, provide a foundation for designing advanced materials, such as liquid-crystalline nanocomposites.^2–5^ By incorporating nanomaterials, it is possible to ensure mesophase stability, enhance existing LC properties, or impart entirely new characteristics to the system.^6–8^ Hybrid systems composed of LCs and nanomaterials, such as metal nanoparticles, have been widely studied in recent decades due to their potential application as tunable metamaterials.^9,10^

One of the nanomaterials of choice for preparing such nanocomposites is gold nanoparticles.^11^ They can be synthesized in a wide range of geometries^12–14^ using well-established methods, which facilitates their selection and optimization for specific applications.^15–17^ Among them, gold nanorods (AuNRs) are particularly attractive due to their shape- and environment-dependent optical properties,^18^ which can be efficiently tuned with the orientational order of LCs.^8,19^ By coupling the anisotropic optical properties of AuNRs, such as localized surface plasmon resonance and polarization sensitivity, with LC mesophases that can be manipulated by external stimuli, these materials allow for large-scale spatial control of nanostructures.^20,21^ The matrix elasticity forces parallel rod alignment along the director. Consequently, these reconfigurable nanocomposites show strong potential for 3D metamaterials with negative refractive indices,^22–24^ subdiffraction-limit superlenses,^25^ and adaptive photonic devices.^26–28^ Depending on the host phase, there are many reports on doping lyotropic,^29–32^ nematic,^33–36^ smectic,^37,38^ chiral,^39–41^ or blue phases^42,43^ with AuNRs. For instance, electric field-induced reorientation of AuNRs in nematic and cholesteric hosts has been demonstrated, enabling tunable optical transmission.^44^ Particular attention has also been paid to their location in defects^36,38^ and the importance of surface functionalization.^45^

In addition to optical tuning, AuNRs can improve the thermal stability of the mesophase, as their shape allows them to fit within the LC structure without disturbing its order.^32^ This stability can also be influenced by differences in the free volume of the matrix, as various nanoparticles can affect molecular packing and chain mobility. Their appropriate shape and size allow them to integrate effectively within the LC domains, maintaining the overall integrity of the composite.^46^

One of the well-known, versatile anionic surfactants that form lyotropic LC phases is sodium bis­(2-ethylhexyl) sulfosuccinate (AOT), which can also form hexagonal phases without the addition of a solvent.^47^ In AOT-based systems, a variety of phases can be achieved, including lamellar, cubic, and inverted hexagonal mesophases.^48^ An AOT-based model mesophase has already been used to determine the positioning and dynamics of metal nanoparticles in the lamellar mesophase, depending on their hydrophilic or hydrophobic properties.^49^ Within mesophases, nanoparticles can be selectively localized in aqueous domains, hydrophobic regions, or defects, depending on their surface functionalization and the physicochemical properties of the host matrix.^46,50–52^ In this context, chemical affinity governs nanoparticle localization while matrix geometry dictates their orientation.

This work presents a detailed nanostructural analysis and assesses the alignment of AuNRs within the liquid crystalline phase template. We focus on the highly regular inverted hexagonal phase (H_2_), which appears at high AOT and low water concentrations.^48^ H_2_ is characterized by long cylindrical water channels arranged in a periodic two-dimensional hexagonal lattice. Theoretical studies on such a mesophase have shown the possibility of fabricating hyperbolic metamaterial by doping it with anisotropic and plasmonic nanomaterials.^53^

## Experimental Section

### Polarizing Microscopy

The mesophases were characterized using polarizing microscope Leica Visoria M + P at 23 °C and 530 nm full-wave retardation plate. A drop of a sample was placed on a glass slide with approximately 30 μm spacer, covered with second, covering slide quickly and left overnight for evaporation at 20 °C.

### Inverse Hexagonal Phase Preparation

Sodium bis­(2-ethylhexyl) sulfosuccinate (AOT) and *p*-xylene were purchased from SigmaAldrich. An aqueous dispersion of gold nanorods (AuNRs)­served as the water phase in the ternary system. The targeted mixture proportion was: 70% wt. AOT/4% wt. water/26% wt. *p*-xylene. This proportion allows for easier sample preparation, as AOT is well soluble in organic solvents compared to water. The organic mixture was sonicated for about 30 min to obtain a homogeneous, viscous fluid. After that, the water phase was added, and the resulting mixture was sonicated and vortexed repeatedly until the colorful water phase dispersed completely, then left to stabilize for 24 h in the fridge (5 °C).

### Nanoparticle Synthesis

AuNRs stabilized with cetyltrimethylammonium bromide (CTAB) were synthesized based on the established protocol of seed-mediated synthesis.^54^ The average dimensions of the obtained anisotropic nanoparticles were 63.2 ± 9.2 × 10.4 ± 1.0 nm, with an average aspect ratio (AR = length/width) of 6.1 ± 1.0. The concentration of AuNRs used in nanocomposite preparation was 0.029 nM (0.0016 mg/mL). AuNRs were purified from CTAB excess via centrifugation at 12050 RCF for 7 min twice.

### Multiphoton Microscopy

The images were acquired using a 40x immersion objective (HC APO L U-V-I 40×/0.80 WATER) on a Leica Stellaris 8 DIVE multiphoton microscope (Leica Microsystems GmbH, Mannheim, Germany), equipped with Insight X3 femtosecond laser for two-photon excitation (Spectra-Physics, Santa Clara, California, USA). The transmitted-light images were acquired using polarization contrast and a Leica K5 sCMOS camera at a resolution of 1024 × 1024 (pixel size 310 nm). The fluorescence images were acquired using a 1045 nm fixed laser beam; the emission range on the NDD detector was 550 nm- 800 nm; pixel size was 263 nm in XY; *z*-stack step was 799 nm.

### Transmission Electron Microscopy

Transmission electron microscope Talos F200i (Thermo Fisher Scientific) was operated at an accelerating voltage of 200 kV in STEM mode with a HAADF collection angle of 41–200 mrad and a camera length of 160 mm. The liquid-crystalline sample was placed on a glow-discharged (20 s, 25 mA) Cu grid (Agar Scientific) covered with approximately 25 nm amorphous carbon. A very small amount of the sample was placed on the substrate using a side of a needle and left to evaporate overnight.

## Results and Discussion

Structural arrangement in H_2_ phase is driven by the affinity of AOT polar headgroups for water (Figure 1a) and by hydrophobic interactions within a continuous organic matrix. The initial mixture of AOT, *p*-xylene, and a water solution of cetyltrimethylammonium bromide (CTAB)-covered AuNRs was prepared in 70/26/4% wt. proportions, respectively. *p*-Xylene evaporates faster at room temperature than water, leading to the formation of the H_2_ phase (Figure 1b).^48,55^ The samples were dried at room temperature overnight and left unsealed; however, some residual water may have remained in the structure. The AuNRs used for material preparation are 63 ± 9 nm in length and 10 ± 1 nm in diameter. With an average aspect ratio of 6.1 ± 1.1, the NRs show a high degree of anisotropy (Figure 1c, TEM image). They exhibit a characteristic localized surface plasmon resonance peak in the near-infrared at approximately 1035 nm (Figure 1c), making them a suitable fluorescent marker for excitation wavelengths used in standard multiphoton microscopy. The concentration of AuNRs was 0.029 nM, providing dense NRs packing within the LC matrix. Given the low water content in the H_2_ mesophase, the approximate water channel diameters are 3–5 nm according to the literature.^56^ As AuNRs used in this research have a diameter of 10 ± 1 nm we assume that the dimensions of the water channels restrict their cross-sectional width to accommodate only a single AuNR (represented graphically in Figure 1d). The discussion on the alignment mechanism and diameter mismatch is provided in the further part of the article.

**Figure 1:**
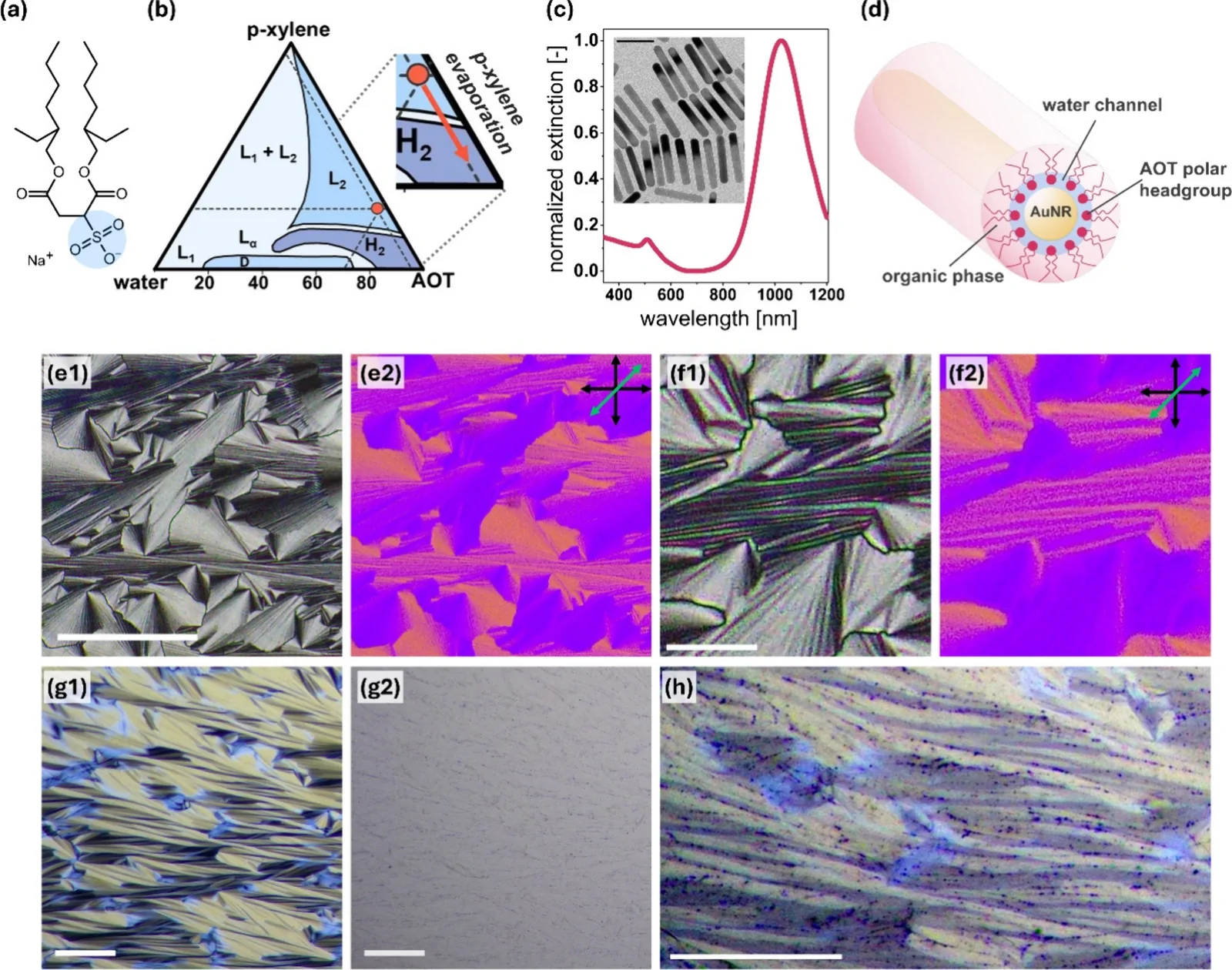
(a) AOT molecule with the highlighted hydrophilic sulfonate group; (b) simplified phase diagram of ternary AOT/*p*-xylene/water system. The diagram was adapted with permission from the reference^48^ (Copyright 1970 Elsevier Inc.); L_1_ and L_2_ – microemulsions, L_α_ – lamellar phase, H_2_ – inversed hexagonal phase, D – bicontinious cubic phase; (c) UV-Vis-NIR spectrum of AuNRs with the corresponding TEM image, the scale bar is 50 nm; (d) Graphical presentation of 10 nm – diameter gold nanorod embedded in water channel inside the inversed columnar phase; (e1,e2) PLM image of H_2_ -AuNRs 30 μm thick composite formed upon solvent evaporation under standard crossed polarized light conditions (e1) and with the insertion of λ-waveplate (e2), the slow axis direction is marked by green arrow; (f1, f2) PLM image of magnified area from e1 and e2 representing a typical textures of green-purple stripes and fan-like regions; (g1) PLM view of 70 μm sample and (g2) corresponding standard bright-field image without the polarizers showing the elongated aggregates; (h) magnified view of g1 and g2 merged with increased g2 contrast. The scale bar represents 100 μm for (e1-h).

Polarized light microscopy (PLM) imaging of the 30 μm thick H_2_–AuNRs nanocomposite shows regular, twisted lines oriented in one direction, separated by regular fan-like regions (Figure 1e1,e2), which is a typical texture for the hexagonal phase.^57,58^ While individual water channels cannot be resolved by PLM, their collective arrangement is reflected in the observed textures: linear domains suggest a preferred parallel orientation of the channels, whereas fan-like regions correspond to radially oriented domains. Moving toward the center of the droplet, the texture transitions into a less ordered, fan-like morphology (Figure S1). In contrast, the regions closer to the droplet edge exhibit a highly ordered structure, characterized by parallel alternating columns of distinct green and purple stripes (Figure 1f1,f2). As the H_2_ phase exhibits negative birefringence,^59^ the blue regions in Figure 1e2,f2 correspond to water columns oriented parallel to the slow axis of the waveplate (presented by the green arrow in the images), while the yellow regions correspond to the perpendicular orientation. As the linear domains are aligned nearly parallel to the polarizer, they appear as alternating purple-magenta and orange-yellow bands. This color variation indicates that the domains are not perfectly straight, but rather twist or bend slightly, changing their angle relative to the waveplate. For the thicker 70 μm H_2_–AuNRs nanocomposite (Fig. 1g1), the content of self-ordered AuNR array is sufficiently high to be visible in the transparent LC matrix when the polarizers are removed (Figure 1g2). The aggregates clearly follow the orientation of the H_2_ phase, accumulating both at the boundaries and within specific domains (Figure 1h). However, the transmitted-light image of the AuNR arrays does not provide information on the packing of the AuNRs along the thickness (*z*-axis).

The plasmonic nature of AuNRs enables strong interactions with electromagnetic radiation and provides a tunable optical response. Driven primarily by the strong local electric-field enhancement at their surface plasmon resonance, AuNRs exhibit two-photon luminescence (2PL) under near-infrared excitation, enabling high-resolution 3D imaging deep within thick samples.^17,60,61^ To characterize the H_2_–AuNRs microstructure along the *z*-axis, we performed 3D multiphoton imaging (Leica Stellaris 8 DIVE multiphoton microscope, Leica Microsystems GmbH). A representative area of a typical H_2_ texture was selected for 3D imaging (Figure S2). From the 2PL signal, we can observe AuNR alignment, especially near the domain boundaries (Figure 2a), with visible ordering in the *xy* plane (Figure 2b). Despite this *xy* alignment, the strong 2PL signal in an orthogonal cross-sectional view (Figure 2c,d, see Figure S3 for single full scans) indicates strong AuNRs accumulation directed along the *z*-axis. On the basis of the observed 3D orientational order, characterized by a preferential alignment along the out-of-plane *z* direction, we deduce that the weaker in-plane *xy* ordering is related to the absence of strong anchoring forces (Figure 2e). Therefore, the untreated substrates were insufficient to promote the macroscopically uniform planar orientation of the mesophase. This accumulation of AuNRs along the *z*-axis is driven by evaporation-induced concentration gradients that propagate through the sample’s thickness. As *p*-xylene and water evaporate, the localized shrinking of the H_2_ matrix near the glass slide surface generates mechanical stress and hydrodynamic shear flows. These forces could direct the AuNRs along the vertical gradient, moving them toward the more highly hydrated and lower-viscosity domains within the center of the sample volume.

**Figure 2:**
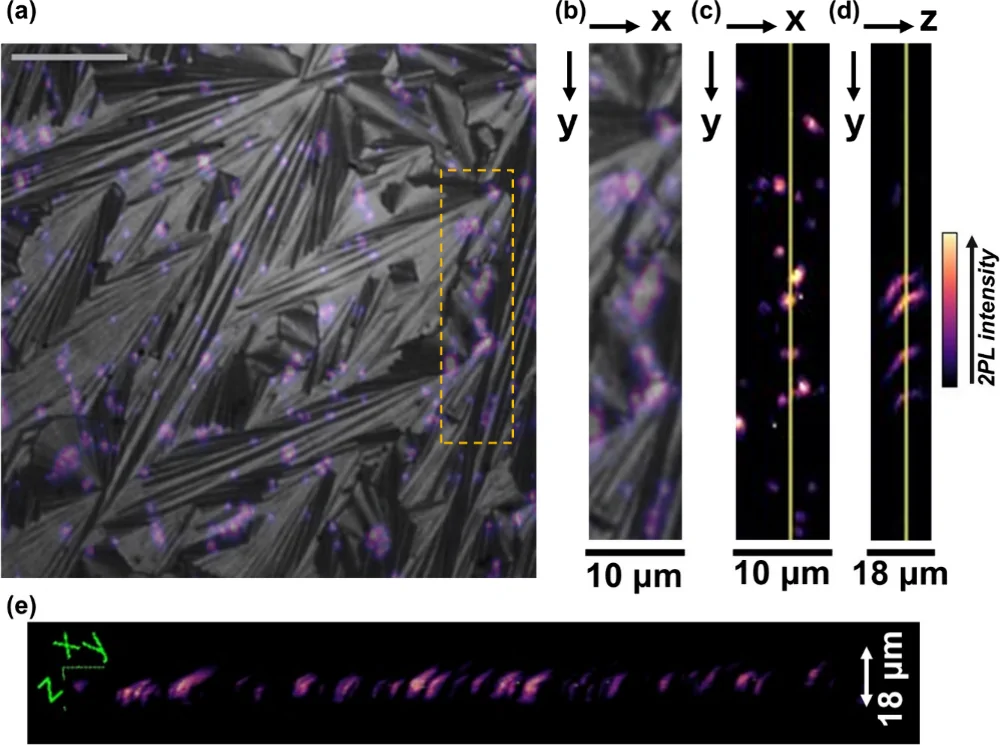
Multiphoton 3D imaging of H_2_–AuNRs nanocomposite. (a) PLM image overlaid with merged *z*-projection image (merged from all *z*-slices). The scale bar is 20 μm; (b) The magnified view indicated by the yellow box in (a); (c) In-plane *xy* 2PL signal of the area indicated in (b), at the *z* = 11.2μm; (d) corresponding orthogonal *yz* cross-sectional view of the same region; (e) 3D projection of the sample cross section, the total 3D scan thickness was 18 μm.

To examine the exact ordering of AuNRs at the nanoscale, we performed STEM imaging (Talos F200i, Thermo Fisher Scientific) of the material on amorphous carbon-covered Cu grid (Figure S4). It should be noted that the sample for STEM imaging undergoes complete dehydration as the microscope operates in a high-vacuum environment (in the order of 10^–7^ Pa). The area near the drop edge (Figure 3a) was thin enough to be electron-transparent. Long, micrometer-sized, ordered AuNRs strings were already visible at higher magnification (Figure 3b). The ordered strings lay parallel to each other throughout the observation area. The magnified microstructure in Figure 3c shows a 15 μm segment of a nearly 36 μm continuous feature. The AuNRs are regularly aligned parallel to the director, forming ordered arrays up to a dozen NRs wide. The total width of the structure is approximately 420 nm (Figure 3d). Some AuNRs overlap, indicating the formation of a 3D nanostructure consisting of several stacked NRs, thereby confirming the formation of the H_2_ mesophase in that region. AuNRs that are oriented at high angles relative to the director indicate structural defects, likely due to the absence of anchoring forces at the top of the sample.

**Figure 3:**
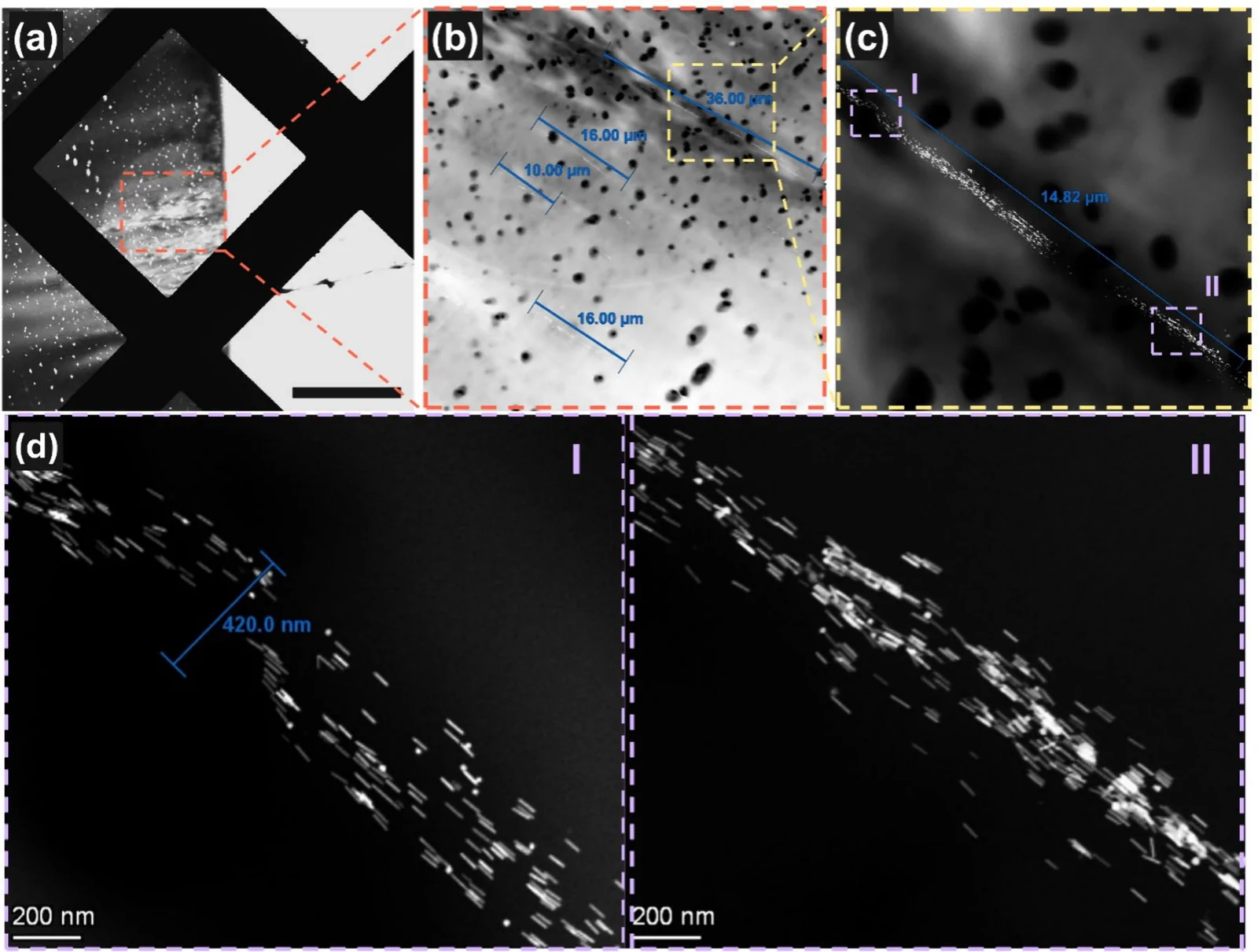
STEM images of thin H_2_–AuNRs nanocomposites. (a) The selected area (orange rectangle) for near-edge area examination. The scale bar is 50 μm. (b) A magnified view of the electron-transparent section. (c) A magnified view of the selected part (the yellow rectangle) of the longest microstructure. (d) Nanoscale view of the two regions indicated by purple rectangles in (c).

Further analysis of the assemblies reveals a specific nanorod spacing within these highly ordered regions (Figure 4a). The statistical analysis using intensity profile plots over distinct areas (Figure S5) indicates the median center-to-center distance between the nanorods of *d*_median_ = 14.9 nm (Figure 4b). Subtracting the average nanorod diameter (10.0 nm) from this value results in an average edge-to-edge spacing of 4.9 nm. The literature sizes of the dried and hydrated CTAB layer on AuNRs oscillate between 1.4–3.7 nm,^62^ respectively, and the approximate AOT monolayer in a reversed micelle is 1.4 nm.^63^ These dimensions limit the internanorod separation to at most two dry CTAB (2.8 nm) and AOT (2.8 nm) layers, which sum to approximately 5.6 nm, only slightly higher than the calculated median nanoparticle interspacing of 4.9 nm (Figure 4a,b). These calculations confirm that the water channel can accommodate only a single nanorod in width. The minor difference between the measured spacing (4.9 nm) and the dry surfactant layers (5.6 nm) may be due to shrinkage and interdigitation of CTAB and AOT hydrophobic tails upon water removal. Therefore, the surfactant shell acts as an effective steric barrier, maintaining a uniform ∼5 nm interparticle distance even after complete sample dehydration.

**Figure 4:**
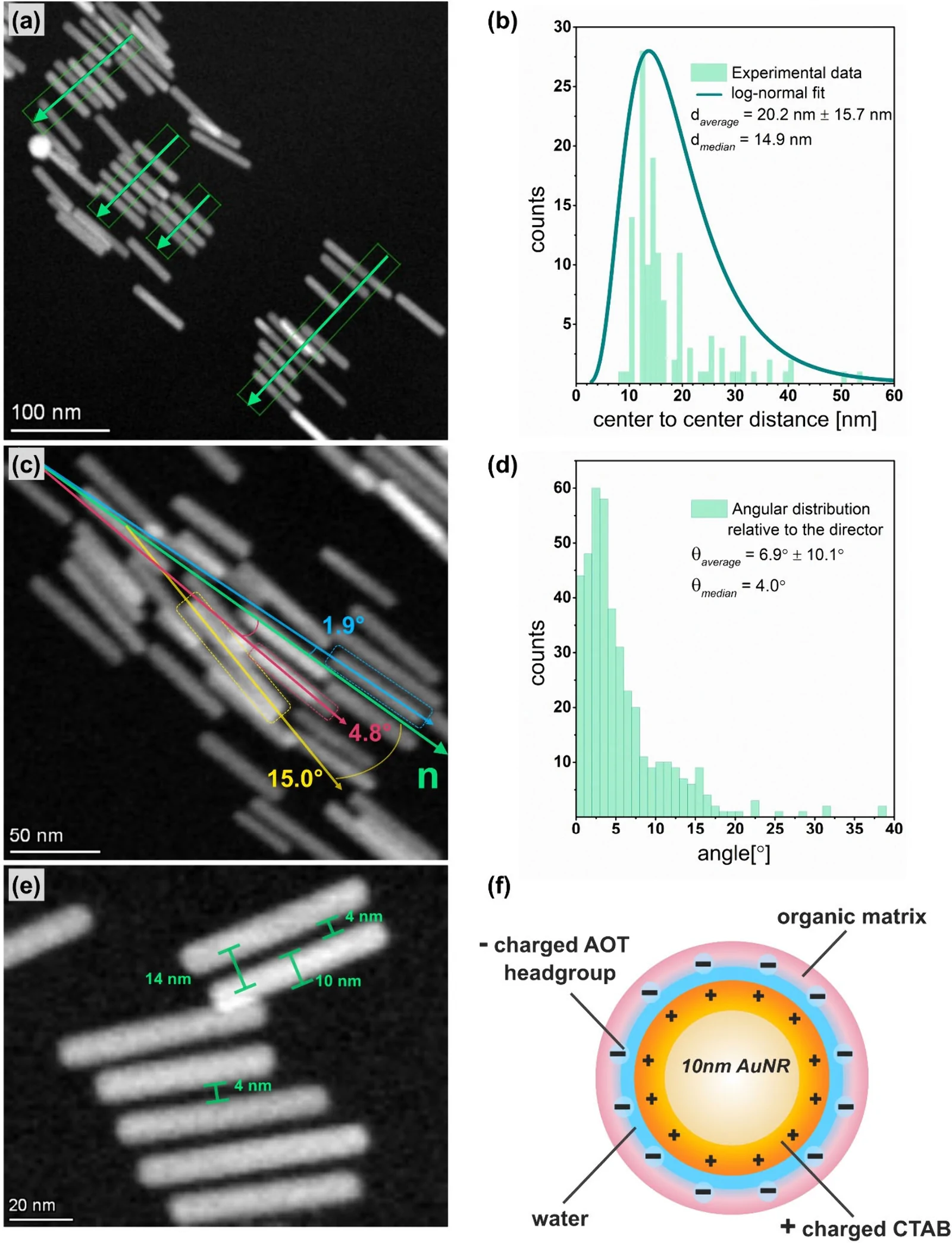
(a) Example region of AuNRs spacing measurement, the arrows indicate the areas used in intensity profiles analysis. (b) Histogram of data from >100 AuNRs spacing measurements; the average value of the center-to-center distance between two nanorods is *d*_avg_ = 20.2 nm ± 15.7 nm and the median value is *d*_median_ = 14.9 nm; The full width at half maximum of log-normal fit equals 13.1 nm. (c) Example area used for measurements of the angle ϑ between the longitudinal axis of individual nanorods (yellow, pink, blue) and the local LC director *n* (green). (d) Histogram representing the angular distribution of AuNRs relative to the director, the average angle is equal to *θ*_avg_ = 6.9 and the median angle is equal θ_median_ = 4.0°. (e) Magnified view on the specific nanoarchitecture with center-to-center and edge-to-edge spacing measurements indicated. (f) Schematic presentation of surfactant layers covering AuNR in water channel of H_2_ phase.

To quantify the accuracy of the alignment of the AuNRs within the H_2_ LC matrix, the angle ϑ between the longitudinal axis of individual nanorods and the local LC director *n* was measured (Figure 4c, see Figure S6 and supplementary information for details). Quantitative orientation analysis revealed a mean alignment angle of θ_avg_ = 6.9 ± 10.1 and the median value of over 400 measurements is equal to θ_median_ = 4.0°. The asymmetric angular distribution, characterized by a sharp peak below 5, indicates strong orientational coupling between the AuNRs and the liquid crystal host (Figure 3d). Because STEM imaging provides a 2D projection of a 3D assembly, these angle measurements cannot determine whether the director or AuNRs exhibit an out-of-plane tilt in the third dimension. Therefore, the 2D orientational order parameter *S*_2D_ was calculated for AuNRs assemblies:^9^

1 $$S_{2D}=2\left\langle \cos^{2}\theta \right\rangle -1$$

The average order parameter calculated for >400 angle measurements equals *S*_2D_ = 0.96 ± 0.10 and for the median θ = 4.0°, the calculated order parameter equals *S*_2D_ = 0.99, which indicates very high degree of AuNRs alignment.

This exceptional ordering of AuNRs was achieved by introducing the nanorods in a highly concentrated aqueous solution at a low volume relative to the AOT content, facilitating the formation of well-ordered, well-separated nanorod assemblies (Figure 4e) within the H_2_ phase. This favorable packing of AuNRs in H_2_O channels is also attributed to attractive electrostatic interactions between the positively charged CTAB on the nanorod surfaces and the negatively charged sulfonate groups of the AOT. The exact behavior of CTAB on the AuNR surface is not yet fully understood,^62,64^ nevertheless, the literature values of zeta potential of CTAB-covered AuNRs clearly indicate the positive charge on the nanomaterial surface.^64–66^ The simplified visualization of AuNR in the hydrated H_2_ phase shows the layered structure, in which the positively charged CTAB layer interacts with the negatively charged AOT headgroups (Figure 4f).

Based on our observations, we propose a dynamic, template-assisted mechanism for the incorporation and alignment of AuNRs in H_2_ matrix. During preparation, in the initial isotropic microemulsion state (L_2_ phase, see Figure 1b), the system has high conformational fluidity. The surfactant molecules and water form a flexible hydration shell surrounding the CTAB-stabilized AuNRs, with no long-range positional or orientational order. According to the literature, the high-aspect-ratio AuNRs can enhance the thermal stability of the H1 phase without disrupting its long-hexagonal order.^32^ As solvent evaporation drives the lyotropic phase transition from the L_2_ phase to H_2_, rather than forcing a rigid 10 nm AuNR into a pre-existing narrow, 3–5 nm water channel, we propose that the surrounding AOT monolayer reorganizes dynamically around the nanorods. This reorganization can be facilitated by the electrostatic interactions between positively charged CTAB and the negatively charged sulfonate headgroup of AOT. The mismatch between native water channels and nanomaterial sizes was already reported in the literature, where an H_2_ matrix was used as a template for anisotropic nanomaterial synthesis, with diameters even up to 30 nm wide,^67,68^ which supports the hypothesis. Previous studies have also shown that nanoparticles can be accommodated within H_2_ phase at low concentration without changing its characteristic symmetry.^69^ Furthermore, the smaller pristine water channels may contribute to the formation of ordered AuNR arrays, as their parallel nucleation around the nanorods guides and stabilizes their long-range alignment according to a mechanism previously demonstrated for AuNRs organization in hexagonal (H_1_) liquid crystal phase.^31^

## Conclusions

In summary, we have demonstrated that our H_2_ template enables dense packing of anisotropic nanomaterials within narrow water channels, directing them into highly ordered arrays. 2D STEM imaging confirmed an exceptional 2D order parameter *S*_2D_ = 0.96 ± 0.10, demonstrating that electrostatic stabilization and surrounding long-range alignment of native water channels drive highly aligned AuNR assembly. In the case of 3D multiphoton imaging, homeotropic alignment of AuNRs was achieved. The cross-sectional *yz*-views indicated a lack of strong lateral alignment along the *y*-direction and an accumulation of AuNRs near the volume center of the 20 μm-thick sample. These findings show that mesophase geometry is a powerful tool for controlling nanoparticle organization. By adding external forces, such as surface anchoring or electromagnetic fields, to guide the LC template, this strategy offers a flexible approach to building plasmonic metamaterials. Ordered nanorod–LCs systems are particularly promising for photonic and optoelectronic applications, because their controlled alignment yields polarization-dependent optical responses and tunable plasmonic coupling.^20,28,31,70^ Because the optical response of AuNRs is highly sensitive to both their spatial organization and the surrounding medium, using an LC template is critical for achieving these outcomes. This developed matrix offers a highly promising platform for the design of advanced functional materials.

## Supplementary Material



## Notes

Additional images of sample overview in low magnification; Multiphoton images of individual slices from 3D *z*-stack; Details on order parameter calculation; intensity profile plots data used for interparticle spacing calculation (PDF)

## References

[1] Bisoyi H. K., Li Q. (2022). Liquid Crystals: Versatile
Self-Organized Smart Soft Materials. Chem. Rev..

[2] O’Neill M., Kelly S. M. (2003). Liquid Crystals
for Charge Transport, Luminescence,
and Photonics. Advanced Materials.

[3] Atorf B., Funck T., Hegmann T., Kempter S., Liedl T., Martens K., Muhlenbernd H., Zentgraf T., Zhang B., Kitzerow H., Urbanski M. (2017). Liquid Crystals and Precious Metal:
From Nanoparticle Dispersions to Functional Plasmonic Nanostructures. Liq. Cryst..

[4] Saadat Y., Imran O. Q., Osuji C. O., Foudazi R. (2021). Lyotropic Liquid Crystals
as Templates for Advanced Materials. J. Mater.
Chem. A Mater..

[5] Lagerwall J., Scalia G., Haluska M., Dettlaff-Weglikowska U., Roth S., Giesselmann F. (2007). Nanotube Alignment Using Lyotropic
Liquid Crystals. Advanced Materials.

[6] Choudhary A., Singh G., Biradar A. M. (2014). Advances
in Gold Nanoparticle-Liquid
Crystal Composites. Nanoscale.

[7] Cseh L., Mehl G. H. (2007). Structure-Property
Relationships in Nematic Gold Nanoparticles. J. Mater. Chem..

[8] Chen C.-H., Dierking I. (2025). Nanoparticles in Thermotropic and Lyotropic Liquid
Crystals. Frontiers in Soft Matter.

[9] Liu Q., Cui Y., Gardner D., Li X., He S., Smalyukh I. I. (2010). Self-Alignment
of Plasmonic Gold Nanorods in Reconfigurable Anisotropic Fluids for
Tunable Bulk Metamaterial Applications. Nano
Lett..

[10] Chen Y., Ai B., Wong Z. J. (2020). Soft Optical Metamaterials. Nano
Converg..

[11] Sardar R., Funston A. M., Mulvaney P., Murray R. W. (2009). Gold Nanoparticles:
Past, Present, and Future. Langmuir.

[12] Chow T. H., Li N., Bai X., Zhuo X., Shao L., Wang J. (2019). Gold Nanobipyramids:
An Emerging and Versatile Type of Plasmonic Nanoparticles. Acc. Chem. Res..

[13] Becerril-Castro I. B., Calderon I., Pazos-Perez N., Guerrini L., Schulz F., Feliu N., Chakraborty I., Giannini V., Parak W. J., Alvarez-Puebla R. A. (2022). Gold Nanostars:
Synthesis, Optical and SERS Analytical
Properties. Analysis & Sensing.

[14] Scarabelli L., Sánchez-Iglesias A., Pérez-Juste J., Liz-Marzán L. M. (2015). A “Tips and Tricks” Practical Guide to
the Synthesis of Gold Nanorods. J. Phys. Chem.
Lett..

[15] Liu K., Zhao N., Kumacheva E. (2011). Self-Assembly of Inorganic Nanorods. Chem. Soc. Rev..

[16] Badir A., Refki S., Sekkat Z. (2025). Utilizing Gold Nanoparticles in Plasmonic
Photothermal Therapy for Cancer Treatment. Heliyon.

[17] Olesiak-Banska J., Waszkielewicz M., Obstarczyk P., Samoc M. (2019). Two-Photon Absorption
and Photoluminescence of Colloidal Gold Nanoparticles and Nanoclusters. Chem. Soc. Rev..

[18] Chen H., Shao L., Li Q., Wang J. (2013). Gold Nanorods and Their
Plasmonic Properties. Chem. Soc. Rev..

[19] Liu Q., Yuan Y., Smalyukh I. I. (2014). Electrically
and Optically Tunable
Plasmonic Guest–Host Liquid Crystals with Long-Range Ordered
Nanoparticles. Nano Lett..

[20] Gardner D. F., Evans J. S., Smalyukh I. I. (2011). Towards
Reconfigurable Optical Metamaterials:
Colloidal Nanoparticle Self-Assembly and Self-Alignment in Liquid
Crystals. Molecular Crystals and Liquid Crystals.

[21] Shen Y., Dierking I. (2019). Perspectives in Liquid-Crystal-Aided
Nanotechnology
and Nanoscience. Applied Sciences.

[22] Kim K., Yoo S., Huh J.-H., Park Q.-H., Lee S. (2017). Limitations and Opportunities
for Optical Metafluids To Achieve an Unnatural Refractive Index. ACS Photonics.

[23] Wang X., Kwon D.-H., Werner D. H., Khoo I.-C., Kildishev A. V., Shalaev V. M. (2007). Tunable Optical
Negative-Index Metamaterials Employing
Anisotropic Liquid Crystals. Appl. Phys. Lett..

[24] Jia D., Yang C., Li X., Peng Z., Liu Y., Cao Z., Mu Q., Hu L., Li D., Yao L., Lu X., Xiang X., Zhang H., Xuan L. (2014). Optical Hyperbolic
Metamaterials Based on Nanoparticles Doped Liquid Crystals. Liq. Cryst..

[25] McLeod E., Ozcan A. (2014). Nano-Imaging Enabled via Self-Assembly. Nano
Today.

[26] Wang M., Yang H., Li X., Zhang X., Yang H. (2025). Self-Assembled
3D Blue Phase Liquid Crystals for Intelligent Photonic Crystals and
Functional Devices. Adv. Opt. Mater..

[27] Ma L.-L., Li C.-Y., Pan J.-T., Ji Y.-E., Jiang C., Zheng R., Wang Z.-Y., Wang Y., Li B.-X., Lu Y.-Q. (2022). Self-Assembled Liquid Crystal Architectures
for Soft Matter Photonics. Light Sci. Appl..

[28] Bisoyi H. K., Li Q. (2022). Liquid Crystals: Versatile
Self-Organized Smart Soft Materials. Chem. Rev..

[29] Fong W.-K., Hanley T. L., Thierry B., Kirby N., Waddington L. J., Boyd B. J. (2012). Controlling the Nanostructure of Gold Nanorod–Lyotropic
Liquid-Crystalline Hybrid Materials Using Near-Infrared Laser Irradiation. Langmuir.

[30] Wang Y. L., Chen L. Y., Liu Q. K., Cai F. H., Qian J. (2016). The Ordering
Alignment of Gold Nanorods in Liquid Crystals and Its Applications
to Polarization-Sensitive SERS. J. Phys. Conf.
Ser..

[31] Liu Q., Cui Y., Gardner D., Li X., He S., Smalyukh I. I. (2010). Self-Alignment
of Plasmonic Gold Nanorods in Reconfigurable Anisotropic Fluids for
Tunable Bulk Metamaterial Applications. Nano
Lett..

[32] Brach K., Matczyszyn K., Olesiak-Banska J., Gordel M., Samoc M. (2016). Stabilization
of DNA Liquid Crystals on Doping with Gold Nanorods. Physical Chemistry Chemical Physics.

[33] Peroukidis S. D., Yannopapas V., Vanakaras A. G., Droulias S., Photinos D. J. (2014). Plasmonic
Response of Ordered Arrays of Gold Nanorods Immersed within a Nematic
Liquid Crystal. Liq. Cryst..

[34] Umadevi S., Feng X., Hegmann T. (2013). Large Area
Self-Assembly of Nematic
Liquid-Crystal-Functionalized Gold Nanorods. Adv. Funct. Mater..

[35] Sridevi S., Prasad S. K., Nair G. G., D’Britto V., Prasad B. L. V. (2010). Enhancement of Anisotropic Conductivity,
Elastic, and
Dielectric Constants in a Liquid Crystal-Gold Nanorod System. Appl. Phys. Lett..

[36] Lee E., Xia Y., Ferrier R. C., Kim H., Gharbi M. A., Stebe K. J., Kamien R. D., Composto R. J., Yang S. (2016). Fine Golden Rings:
Tunable Surface Plasmon Resonance from Assembled Nanorods in Topological
Defects of Liquid Crystals. Advanced Materials.

[37] Milette J., Relaix S., Lavigne C., Toader V., Cowling S. J., Saez I. M., Lennox R. B., Goodby J. W., Reven L. (2012). Reversible
Long-Range Patterning of Gold Nanoparticles by Smectic Liquid Crystals. Soft Matter.

[38] Rožič B., Fresnais J., Molinaro C., Calixte J., Umadevi S., Lau-Truong S., Felidj N., Kraus T., Charra F., Dupuis V., Hegmann T., Fiorini-Debuisschert C., Gallas B., Lacaze E. (2017). Oriented Gold Nanorods and Gold Nanorod
Chains within Smectic Liquid Crystal Topological Defects. ACS Nano.

[39] Chu G., Wang X., Yin H., Shi Y., Jiang H., Chen T., Gao J., Qu D., Xu Y., Ding D. (2015). Free-Standing Optically Switchable Chiral Plasmonic
Photonic Crystal
Based on Self-Assembled Cellulose Nanorods and Gold Nanoparticles. ACS Appl. Mater. Interfaces.

[40] Liu Q., Campbell M. G., Evans J. S., Smalyukh I. I. (2014). Orientationally
Ordered Colloidal Co-Dispersions of Gold Nanorods and Cellulose Nanocrystals. Advanced Materials.

[41] Liu Y., Wei X., Chen Y., Yang Y., Zhang Y., Liu H. (2025). Assembly of
Intrinsic Chiral Gold Nanorods within a Cholesteric Liquid Crystal
Host with Tunable Optical Asymmetry. J. Mater.
Chem. C Mater..

[42] Gandhi S. S., Kim M. S., Hwang J., Chien L. (2016). Electro-optical Memory
of a Nanoengineered Amorphous Blue-Phase-III Polymer Scaffold. Advanced Materials.

[43] Wong J.-M., Hwang J.-Y., Chien L.-C. (2011). Electrically
Reconfigurable and Thermally
Sensitive Optical Properties of Gold Nanorods Dispersed Liquid Crystal
Blue Phase. Soft Matter.

[44] Sheetah G. H., Liu Q., Senyuk B., Fleury B., Smalyukh I. I. (2018). Electric Switching
of Visible and Infrared Transmission Using Liquid Crystals Co-Doped
with Plasmonic Gold Nanorods and Dichroic Dyes. Opt. Express.

[45] Lewandowski W., Wójcik M., Górecka E. (2014). Metal Nanoparticles with Liquid-Crystalline
Ligands: Controlling Metal Nanoparticles with Liquid-Crystalline Ligands:
Controlling Nanoparticle Superlattice Structure and Properties. ChemPhysChem.

[46] Bukowczan A., Hebda E., Pielichowski K. (2021). The Influence
of Nanoparticles on
Phase Formation and Stability of Liquid Crystals and Liquid Crystalline
Polymers. J. Mol. Liq..

[47] Ungar G., Tomašić V., Xie F., Zeng X. (2009). Structure
of Liquid Crystalline Aerosol-OT and Its Alkylammonium Salts. Langmuir.

[48] Ekwall P., Mandell L., Fontell K. (1970). Some Observations
on Binary and Ternary
Aerosol OT Systems. J. Colloid Interface Sci..

[49] Sui Z. M., Chen X., Wang L. Y., Xu L. M., Yang C. J. (2006). Study on
the Doping and Interactions of Metal Nanoparticles in the Lyotropic
Liquid Crystals. Acta Physico - Chimica Sinica.

[50] Liu X., Qi G., Park A. M. G., Rodriguez-Gonzalez A., Enotiadis A., Pan W., Kosma V., Fuchs G. D., Kirby B. J., Giannelis E. P. (2019). Scalable
Synthesis of Switchable Assemblies of Gold Nanorod Lyotropic Liquid
Crystal Nanocomposites. Small.

[51] Olesiak-Banska J., Gordel M., Matczyszyn K., Shynkar V., Zyss J., Samoc M. (2013). Gold Nanorods as Multifunctional Probes in a Liquid Crystalline DNA
Matrix. Nanoscale.

[52] Coursault D., Grand J., Zappone B., Ayeb H., Lévi G., Félidj N., Lacaze E. (2012). Linear Self-Assembly of Nanoparticles
Within Liquid Crystal Defect Arrays. Advanced
Materials.

[53] Li X., Yang C., Jia D., Cao Z., Mu Q., Hu L., Peng Z., Liu Y., Yao L., Lu X., Xuan L. (2013). Wide-Spectrum Optical Hyperbolic Metamaterial Based on Reverse Hexagonal
Lyotropic Liquid Crystal. Opt. Commun..

[54] Chang H. H., Murphy C. J. (2018). Mini Gold Nanorods with Tunable Plasmonic Peaks beyond
1000 Nm. Chem. Mater..

[55] Oswald, P. ; Pieranski, P. Nematic and Cholesteric Liquid Crystals; CRC Press, 2005. 10.1201/9780203023013.

[56] Ramezanpour M., Schmidt M. L., Bashe B. Y. M., Pruim J. R., Link M. L., Cullis P. R., Harper P. E., Thewalt J. L., Tieleman D. P. (2020). Structural
Properties of Inverted Hexagonal Phase: A Hybrid Computational and
Experimental Approach. Langmuir.

[57] Saupe A. (1977). Textures,
Deformations, and Structural Order of Liquid Crystals. J. Colloid Interface Sci..

[58] Jiang W., Yu B., Liu W., Hao J. (2007). Carbon Nanotubes Incorporated within
Lyotropic Hexagonal Liquid Crystal Formed in Room-Temperature Ionic
Liquids. Langmuir.

[59] Oka T., Ohta N. (2016). Two Distinct Cylinder
Arrangements in Monodomains of a Lyotropic
Liquid Crystalline Hexagonal II Phase: Monodomains with Straight Cylinders
and Ringed Cylinders in Capillaries. Langmuir.

[60] Huang X., El-Sayed M. A. (2010). Gold Nanoparticles: Optical Properties and Implementations
in Cancer Diagnosis and Photothermal Therapy. J. Adv. Res..

[61] Brach K., Olesiak-Banska J., Waszkielewicz M., Samoc M., Matczyszyn K. (2018). DNA Liquid
Crystals Doped with AuAg Nanoclusters: One-Photon and Two-Photon Imaging. J. Mol. Liq..

[62] Pedrazo-Tardajos A., Claes N., Wang D., Sánchez-Iglesias A., Nandi P., Jenkinson K., De Meyer R., Liz-Marzán L. M., Bals S. (2024). Direct Visualization of Ligands on Gold Nanoparticles in a Liquid
Environment. Nat. Chem..

[63] Eskici G., Axelsen P. H. (2016). The Size of AOT
Reverse Micelles. J. Phys. Chem. B.

[64] Burrows N. D., Lin W., Hinman J. G., Dennison J. M., Vartanian A. M., Abadeer N. S., Grzincic E. M., Jacob L. M., Li J., Murphy C. J. (2016). Surface Chemistry of Gold Nanorods. Langmuir.

[65] Orendorff C. J., Hankins P. L., Murphy C. J. (2005). PH-Triggered
Assembly of Gold Nanorods. Langmuir.

[66] Murphy C.
J., Thompson L. B., Alkilany A. M., Sisco P. N., Boulos S. P., Sivapalan S. T., Yang J. A., Chernak D. J., Huang J. (2010). The Many Faces
of Gold Nanorods. J. Phys. Chem. Lett..

[67] Huang N. M., Shahidan R., Khiew P. S., Peter L., Kan C. S. (2004). In Situ
Templating of PbS Nanorods in Reverse Hexagonal Liquid Crystal. Colloids Surf. A Physicochem. Eng. Asp..

[68] Huang L. M., Wang H. T., Wang Z. B., Mitra A., Bozhilov K. N., Yan Y. S. (2002). Nanowire Arrays
Electrodeposited from Liquid Crystalline
Phases. Advanced Materials.

[69] Venugopal E., Bhat S. K., Vallooran J. J., Mezzenga R. (2011). Phase Behavior of Lipid-Based
Lyotropic Liquid Crystals in Presence of Colloidal Nanoparticles. Langmuir.

[70] Koenig G. M., Meli M.-V., Park J.-S., de Pablo J. J., Abbott N. L. (2007). Coupling
of the Plasmon Resonances of Chemically Functionalized Gold Nanoparticles
to Local Order in Thermotropic Liquid Crystals. Chem. Mater..

