# Supplementary material for: Nanostructural Characterization of Gold Nanorod Assemblies in Hexagonal Liquid Crystalline Template
Source: Langmuir. 2026 Sep 10;42(37):27514–21. doi: 10.1021/acs.langmuir.6c03954 (PMC13602915; doi:10.1021/acs.langmuir.6c03954)
Supplement: Supplementary file 1 [file la6c03954_si_001.pdf]

SUPPORTING INFORMATION

Nanostructural Characterization of Gold Nanorod Assemblies in Hexagonal Liquid Crystalline Template

Olga Kaczmarczyk[1], Dominika Benkowska-Biernacka[1], Julita Wesołowska[2], Mateusz Grzybowski[1], Amelia Maj[1], Katarzyna Matczyszyn[1], Andrzej Żak[1]*

[1] Institute of Advanced Materials, Wroclaw University of Science and Technology, 27 Wybrzeze Stanislawa Wyspianskiego St., 50-370 Wroclaw, Poland

[2] Laboratory of Microscopic Imaging, Maj Institute of Pharmacology Polish Academy of Sciences, CEPHARES, 12 Smetna St., 31-343 Krakow, Poland

**olga.kaczmarczyk@pwr.edu.pl*

Table of contents

Figure S1. .... **S2**
Figure S2. .... **S3**
Figure S3. .... **S4**
Figure S4. .... **S5**
Figure S5. .... **S6**
AuNRs ordering along director measurement **S7**
Figure S6. .... **S7**

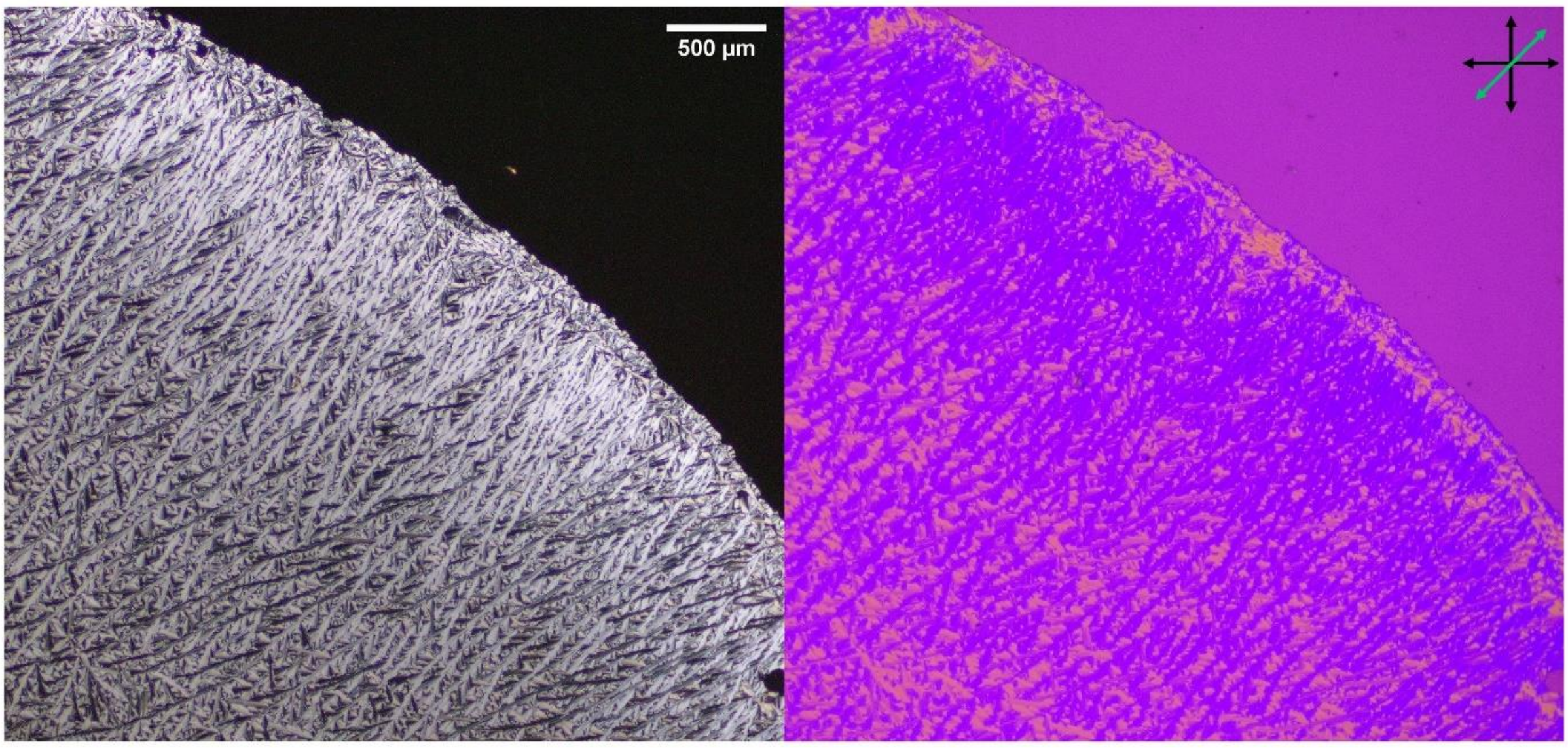

Figure S1. PLM images of the H_2-AuNRs nanocomposite at low magnification showing the high ordering closer to the droplet edge.

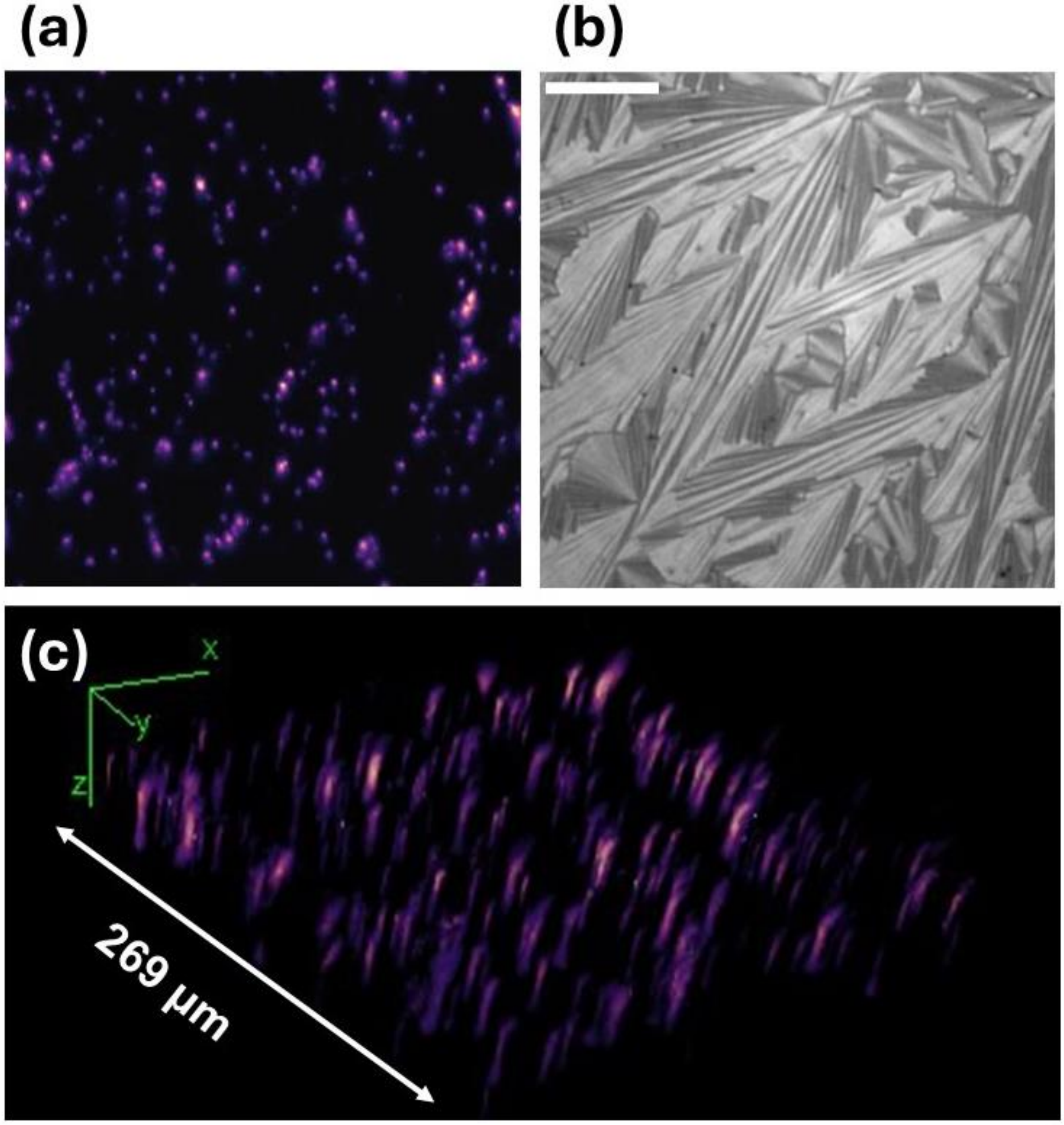

Figure S2. (a) 2D maximum intensity projection of the 2PM z-stack, merged across all collected z-slices; (b) PLM image of the 2PM imaging area. The scale bar is 20 µm; (c) 3D projection of full imaging area.

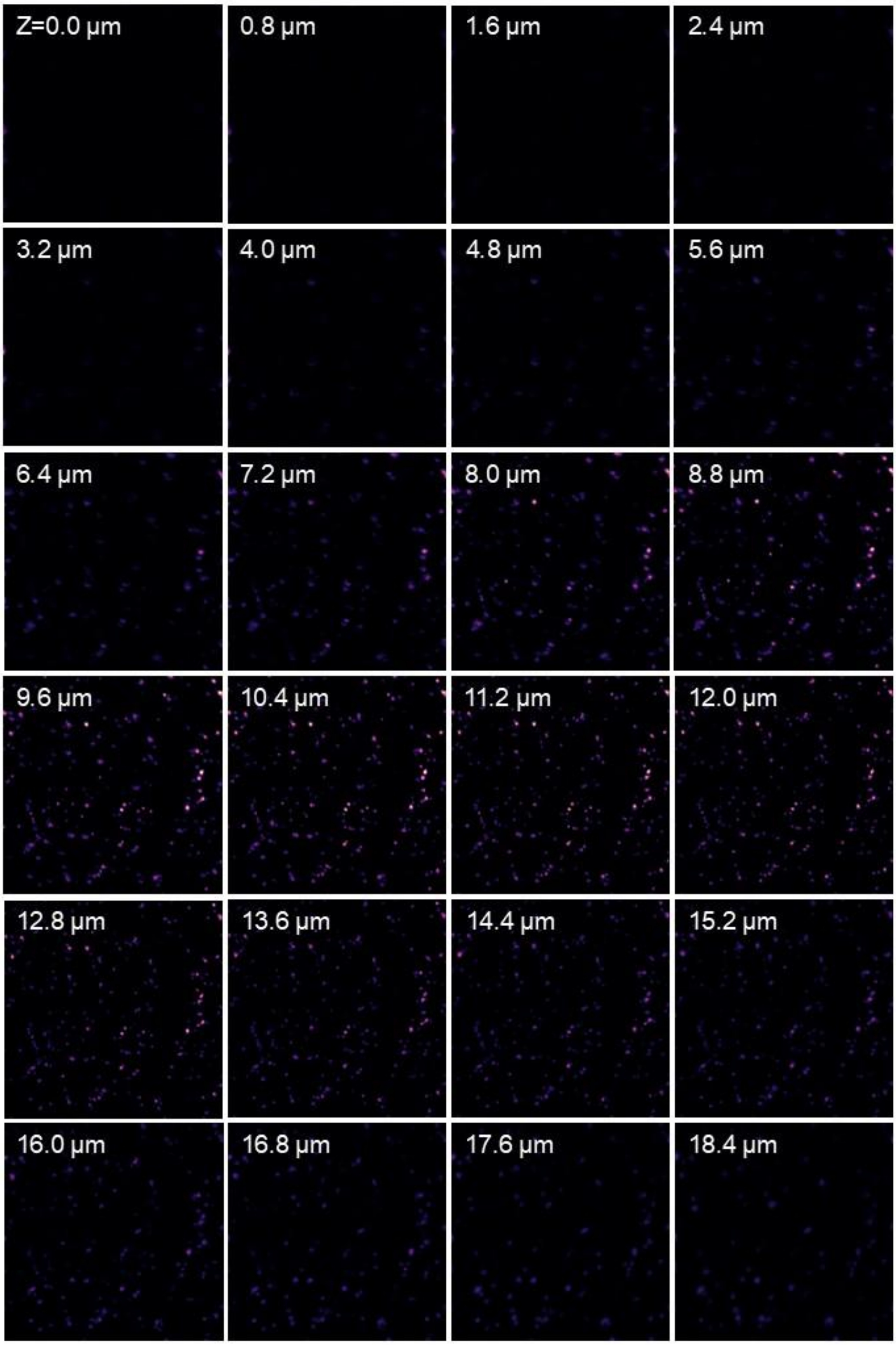

Figure S3. Single slices of measured z-stacks over 18.4 µm in depth. The scan sizes are 269 µm x 269 µm.

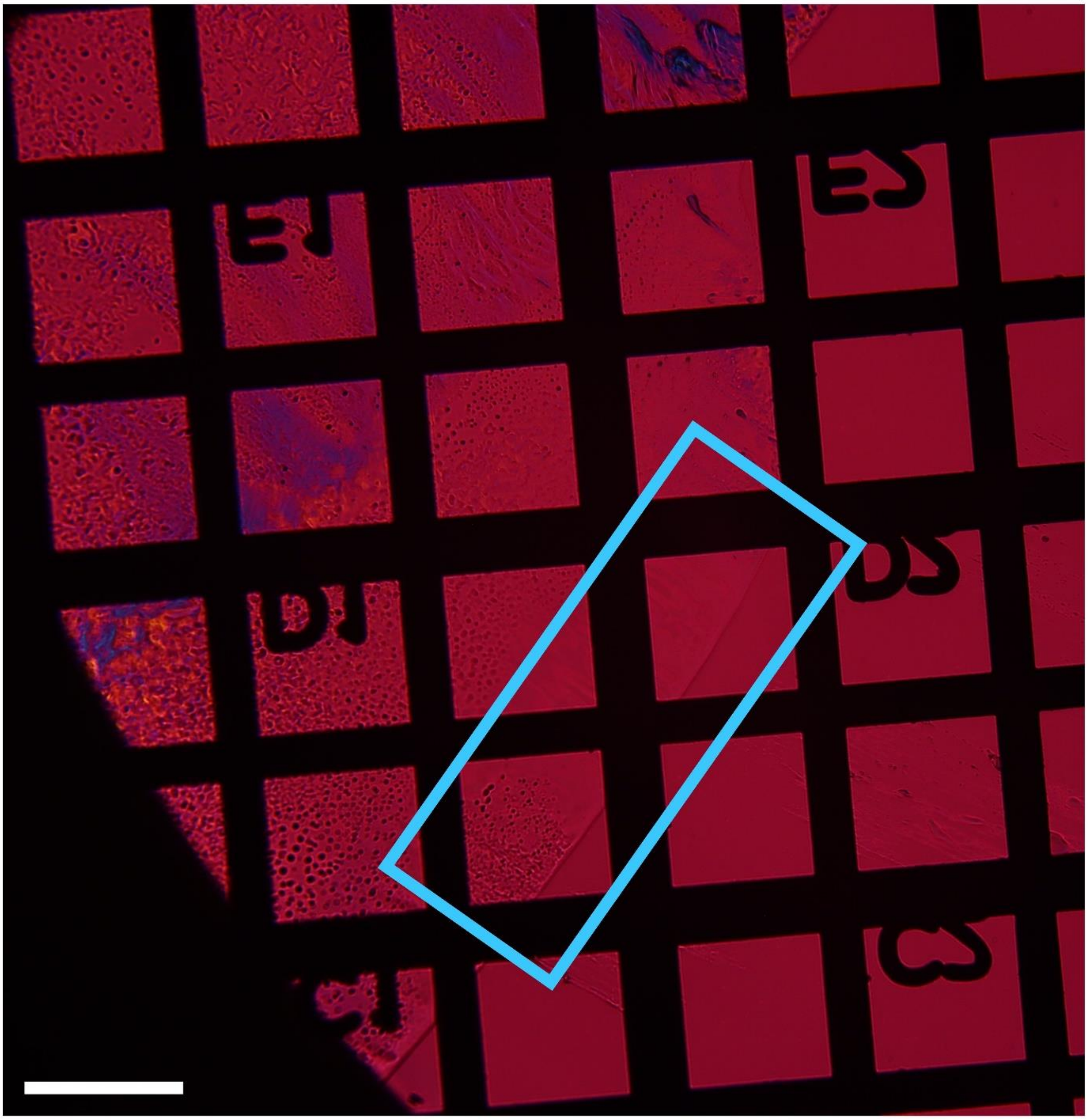

Figure S4. PLM image of TEM grid with the thin drop edge that was characterized using STEM. The scale bar is 100 μm.

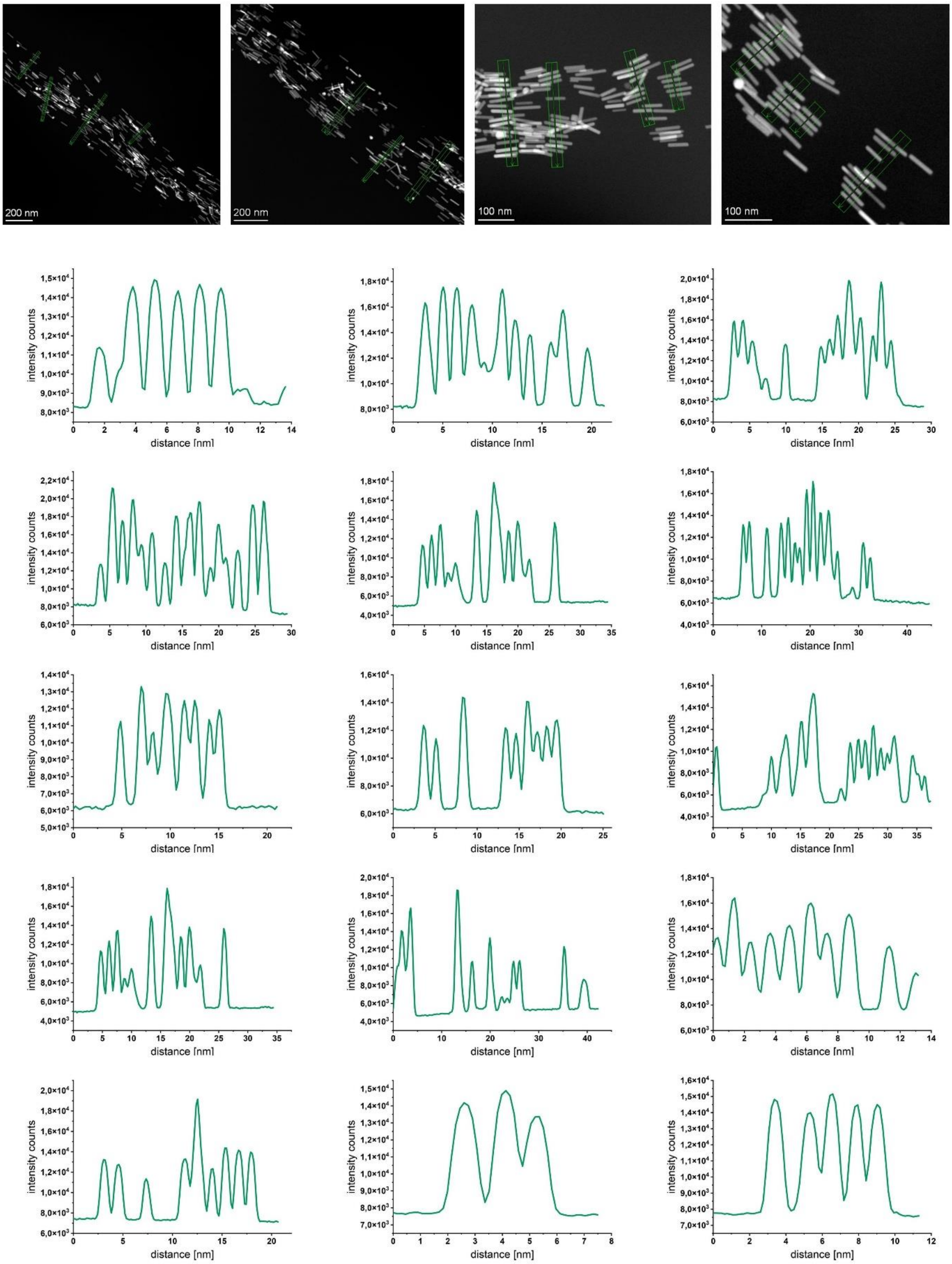

Figure S5. Representative STEM images of highly ordered H_2-AuNRs nanocomposite areas used for interspacing measurements, with the corresponding intensity profile plots. A total of 113 individual interspacing measurements were performed to determine the average spacing.

AuNRs ordering along director measurement

The angle of a nanoparticle relative to the director is the difference between the director angle and the particular nanoparticle angle. Based on an image of the entire measurement area (Fig. 3a), the angle of the director was determined by drawing a line *a* along the director (approximated by the relative direction of the nanoparticle string) (Fig. S6a) and measuring its (absolute) angle. To improve accuracy, angle measurements for individual nanoparticles were obtained from 3 images covering different areas of the director (Fig. S6b). Each (absolute) angle was measured by drawing a line *b* along the length of the nanoparticle (Fig. S6c). All measurements were performed using ImageJ.

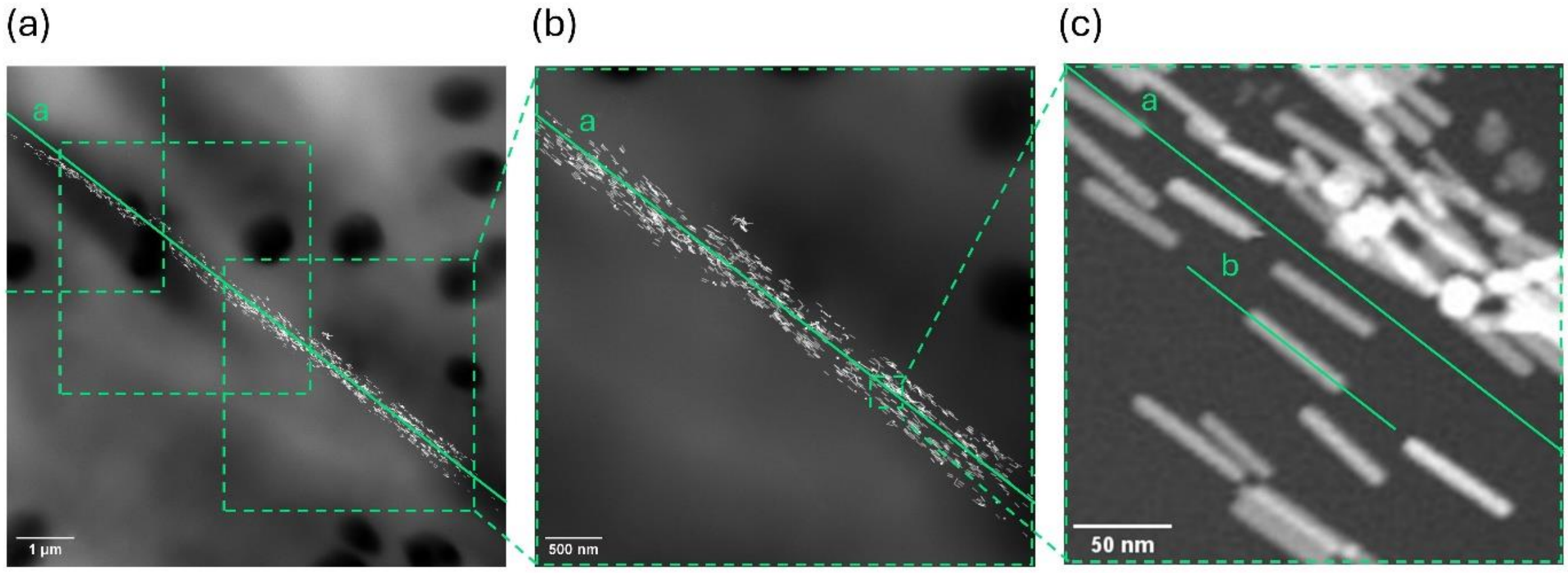

Figure S6. STEM photos used for the director and NPs angle measurements. (a) Image (11.5kx mag.) of the whole measurement area with the director *a* and three images done at higher (23.0kx) magnification *marked*. (b) One of the higher mag. images used for carrying out NPs angle measurements. (c) 23.0kx image with additional ~15x zoom for precise NR angle measurement *b*.
